# Testicular Cancer in Infertile Men With and Without Testicular Microlithiasis: A Systematic Review and Meta-Analysis of Case-Control Studies

**DOI:** 10.3389/fendo.2019.00164

**Published:** 2019-03-21

**Authors:** Arcangelo Barbonetti, Alessio Martorella, Elisa Minaldi, Settimio D'Andrea, Dorian Bardhi, Chiara Castellini, Felice Francavilla, Sandro Francavilla

**Affiliations:** Andrology Unit, Department of Clinical Medicine, Public Health, Life and Environment Sciences, University of L'Aquila, L'Aquila, Italy

**Keywords:** testicular microlithiasis, testicular cancer, germ cell tumor, male infertility, ultrasonography

## Abstract

**Background:** An association between testicular microlithiasis (TM) and both carcinoma *in situ* (CIS) of the testis and testicular germ cell tumors (TGCTs) has been reported. Furthermore, TM seems to be significantly more prevalent in men with male-factor infertility, representing itself a risk factor for TGCT. Nevertheless, the evidence of the association of TM with a higher prevalence of testicular cancer in infertile men remains inconclusive. The aim of this study was to systematically evaluate whether, and to what extent, TM is associated to a significantly higher prevalence of testicular cancer in infertile males.

**Methods:** A thorough search of MEDLINE, SCOPUS, CINAHL, WEB OF SCIENCE, and Cochrane Library databases was carried out to identify case-control studies comparing the prevalence of testicular cancer in infertile men with and without TM. Methodological quality of the studies was assessed using the Newcastle-Ottawa Scale. In the absence of heterogeneity, odds ratios (ORs) with 95% confidence intervals (CIs) for testicular cancer were combined using a fixed effect model. Funnel plots and trim-and-fill analysis were used to assess publication bias.

**Results:** Eight studies met the inclusion criteria and provided information on 180 infertile men with TM and 5,088 infertile men without TM. The pooled OR indicated that the presence of TM is associated with a ~18-fold higher odd for testicular cancer (pooled OR:18.11, 95%CI: 8.09, 40.55; *P* < 0.0001). No heterogeneity among the studies was observed (*P*_for heterogeneity_ = 0.99, *I*^2^ = 0%). At the sensitivity analysis, similar pooled ORs and 95%CIs were generated with the exclusion of each study, indicating the high degree of stability of the results. The funnel plot revealed a possible publication bias and the trim-and-fill test detected two putative missing studies. Nevertheless, even when the pooled estimate was adjusted for publication bias, there was a still significantly higher odd for testicular cancer in the TM group (adjusted pooled OR: 16.42, 95%CI: 7.62, 35.37; *P* < 0.0001).

**Conclusions:** In infertile men the presence of TM is associated to an ~18-fold higher prevalence of testicular cancer. Longitudinal studies are warranted to elucidate whether this cross-sectional association actually reflects a higher susceptibility of infertile men with TM to develop testicular cancer over time.

## Introduction

Testicular microlithiasis (TM) usually represents an incidental finding during a scrotal ultrasonography (US) examination which shows a typical speckled pattern of the testicular parenchyma with multiple, tiny, bright non-shadowing echogenic foci, involving one or both testes, due to intratubular microcalcifications (1).

Testicular microlithiasis in itself does not represent a malignant condition and, in different series of patients referred for urologic evaluation, albeit infrequent, it has been found in association with a number of non-neoplastic disorders such as cryptorchidism (2–4), epididymitis (5, 6) and testicular torsion (5, 7). Nevertheless, as suggested by some authors, TM should be regarded as a visible sign of a premalignant condition, since an association between TM and both carcinoma *in situ* (CIS) of the testis (3, 8–10) and testicular germ cell tumors (TGCTs), i.e., seminomas and non-seminomas (5, 6, 11–17), has been reported.

The relationship between TM and testicular cancer would be of special concern in infertile male population, as male-factor infertility in itself has been associated with an increased risk of TGCT (18, 19). In particular, in a large retrospective cohort study by Hanson et al. (19), men with oligozoospermia had a >10-fold increase in the risk of testicular cancer when compared to fertile men. Indeed, TM and male-factor infertility due to poor spermatogenesis, together with other closely related clinical conditions, such as cryptorchidism and urogenital malformations, could share common pathogenetic mechanisms related to testicular dysgenesis syndrome (20). This could explain the reported higher prevalence of TM in infertile men populations when compared to men referred for scrotal complaints or young asymptomatic males (21, 22).

However, whether and to what extent the presence of TM in infertile men actually confers a significantly higher risk of testicular cancer remains unclear, as, in this population, an association of TM with a higher prevalence of testicular cancer has been reported by some studies (23–26) but not by others (2, 7, 27, 28).

Hence, we carried out a systematic review with meta-analysis of the available case-control studies, aiming to answer the following question: “Does, and to what extent, TM is associated to a significantly higher prevalence of testicular cancer in infertile males?”

## Materials and Methods

The study was conducted according to the Cochrane Collaboration and the Preferred Reporting Items for Systematic reviews and Meta-Analyses (PRISMA) statement (29). It also complies with the guidelines of Meta-Analyses and Systematic Reviews of Observational Studies (MOOSE) (30). PRISMA and MOOSE Checklists have been presented as Supplementary Tables 1, 2.

The study is registered in the International Prospective Register of Systematic Reviews (PROSPERO) with the registration number CRD42019121488.

### Systematic Search Strategy

We conducted a systematic search in MEDLINE, SCOPUS, CINAHL, WEB OF SCIENCE, and Cochrane Library databases to identify all relevant studies in the English language with the terms: (“testicular microlithiasis”) AND (“testicular cancer” OR “testicular tumor^*^” OR “testicular neoplasm^*^” OR “germ cell cancer” OR “germ cell tumor^*^” OR TGCT OR “germ cell neoplasm^*^” OR seminoma^*^ OR nonseminoma^*^). If it was not clear from the title and abstract whether the paper contained relevant data, the full paper was retrieved. We scrutinized the reference lists of the identified articles to find possible additional pertinent studies.

### Inclusion and Exclusion Criteria

The outcome of interest was a difference in the prevalence of testicular cancer between infertile men with and without TM. The eligibility criteria used for the inclusion were: (1) observational case-control studies involving adult men undergoing scrotal US as a part of diagnostic work-up for infertile marriage with (cases) and without (controls) TM; (2) availability of data for the calculation of odds ratios (ORs) with a 95% confidence interval (CI) for testicular cancer in both the groups. As variable ultrasonographic definitions of TM have been reported in literature, no restrictions in diagnostic criteria for TM were used when assessing the eligibility of the studies.

Two independent reviewers (AM and EM) assessed the eligibility of each selected article and any disagreement was resolved via discussion involving a third reviewer (AB).

### Data Extraction

Data from the selected articles were extracted by including the first author, publication year, country, the total number of cases (infertile men with TM) and controls (infertile men without TM), and the number of events (number of patients with testicular cancer) in each group. Additional information, when available, included: mean age or age range of the participants, testicular volumes, semen characteristics, and the percentage of cases with bilateral TM and/or cryptorchidism history. Wherever quantitative data were missing or inconsistent, the authors were contacted to obtain the necessary information.

### Quality Assessment

The quality of studies included in the quantitative analysis was assessed using the “star system” of the Newcastle-Ottawa Quality Assessment Scale (NOS) (31). The minimum score was 0 stars and the maximum that could be awarded was 9 stars. Studies getting scores ≥6 stars were regarded as good quality studies. The quality assessment was performed by two reviewers (AB and SDA) and any disagreement was resolved by a third reviewer (SF) who re-evaluated the original study.

### Statistical Analysis

The relationship between TM and testicular cancer was assessed using ORs and a 95% CI as well as by Mantel-Haenszel estimates. In the absence of heterogeneity between the studies, data were combined using a fixed effect model. The Cochrane Chi-square (Cochrane Q) test and the *I*^2^ test were carried out to analyze the heterogeneity between the results of different studies. An *I*^2^ > 50% and/or *P* < 0.05 indicated substantial heterogeneity (32).

Sensitivity analysis was performed by sequential omission of individual studies to determine the contribution of each study to the pooled estimate and evaluate the stability of the results.

Publication bias was graphically identified using a funnel plot, wherein a symmetric inverted funnel shape arises from a “well-behaved” data set, in which publication bias is unlikely (33). The funnel plot was also subjected to the Duval and Tweedie's “trim-and-fill” analysis, which, in the presence of asymmetric shape, detects putative missing studies to rebalance the distribution. This analysis also provides an adjusted pooled estimate taking the additional studies into account, thus correcting the analysis for publication bias (34).

The extracted data were analyzed using the package “metafor” of the statistical software R (version 3.0.3; R Foundation for Statistical Computing, Vienna, Austria).

## Results

### Study Selection

**Figure 1:**
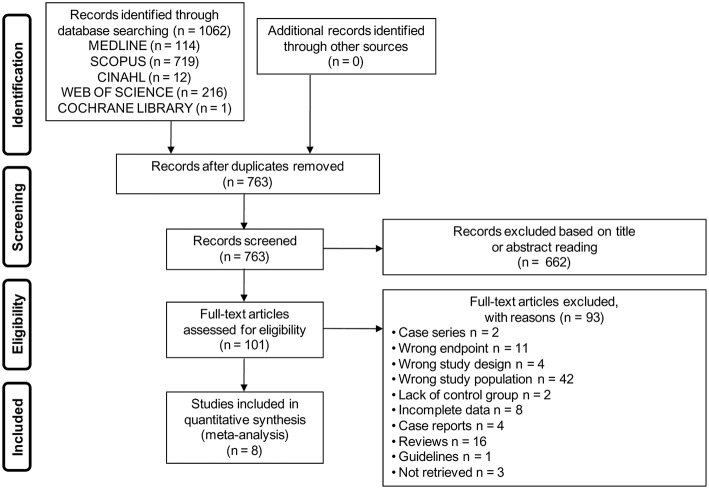
Flow diagram showing an overview of the study selection process.

The electronic search yielded a total of 1,062 studies. After removal of duplicate, 763 studies were left, of which 662 were excluded based on titles and abstracts. Hence, as shown in Figure 1, a total of 101 studies were identified, of which 8 met the inclusion criteria (2, 7, 23–28).

Main details of the articles included in the quantitative synthesis are reported in Table 1.

**Table 1:** Main characteristics of the eight studies included.

**Study**	**Region**	**Mean age or age range of participants (years)**	**TM group *n* (%)**	**Confirmation of cancer diagnosis after US**	**Bilateral testicular volume (mean ± SD)**	**Cryptorchidism history (%) in TM group**	**Bilateral TM (%)**	**Semen abnormalities**
Aizenstein et al. (2)	USA	37	5 (2.8)	NA	NR	40	50	All participants had oligo and/ or astheno
La Vignera et al. (26)	Italy	43.3	60 (18.8)	Histology	NR	NR	NR	Oligo/astheno and/or terato in 66.6% of the TM group
Mazzilli et al. (27)	Italy	NA	13 (4.6)	NA	Whole population: 18.0 ± 4.5 ml	NR	NR	In the whole study population: oligo: 23.8% azo: 5.6% astheno: 70.4% terato: 20.3%
Negri et al. (25)	Italy	37	31 (1.4)	Histology	NR	13	NR	NS
Pierik et al. (23)	NL	20–58	12 (0.9)	Histology	NR	33.3	NR	NS
Qublan et al. (28)	Jordan	31	23 (9.8)	NA	Whole population: 14.0 ± 3.6 ml	NR	NR	All participants had oligo or azo
Sakamoto et al. (24)	Japan	35.8	31 (5.6)	Histology	TM group: 9.0 ± 5.2 ml Controls: 9.8 ± 5.1 ml	3.2	90.3	Oligo or azo in 64.5% of whole study population
Thomas et al. (7)	UK	29–51	5 (3.1)	NA	NR	0	0	NS

*Astheno, asthenozoospermia; Azo, azoospermia; Oligo, oligozoospermia; Terato, teratozoospermia; TM, testicular microlithiasis; NA, not applicable (no testicular masses were detected at US); NL, Netherlands; NR, not reported; NS, not specified; SD, standard deviation; US, ultrasonography*.

### Quality of the Included Studies

The NOS score-based quality ratings of the studies are presented in Table 2. Quality scores ranged from 3 to 7. Seven articles were considered to be of good quality (2, 7, 24–28) scoring ≥6 and one article (23) was assessed to be of poor quality. In particular, in all studies except that by Pierik et al. (23), diagnostic criteria for TM used for the definition of cases were clearly reported: according to Backus et al. (8), TM was defined as ≥5 randomly distributed non-shadowing hyperechogenic foci with diameters <3 mm per transducer field. In most studies a full comparability could not be ensured by adjusting either on age or other variables (Table 2).

**Table 2:** Newcastle-Ottawa assessment scale for case-control studies.

**Study**	**Selection**					**Comparability**			**Exposure**			**Total**
	**Definition of cases**	**Representa- tiveness of cases**	**Selection of controls**	**Definition of controls**		**On age**	**On other risk factors**		**Assessment of exposure**	**Same methods of ascertain- ment for cases and controls**	**Non response rate**	
Aizenstein et al. (2)	1	1	1	1		0	0		1	1	0	6
La Vignera et al. (26)	1	1	1	1		0	0		1	1	0	6
Mazzilli et al. (27)	1	1	1	1		0	1		1	1	0	7
Negri et al. (5)	1	1	1	1		0	0		1	1	0	6
Pierik et al. (23)	0	1	1	0		0	0		1	0	0	3
Qublan et al. (28)	1	1	1	1		0	0		1	1	0	6
Sakamoto et al. (24)	1	1	1	1		1	1		1	0	0	7
Thomas et al. (7)	1	1	1	1		0	0		1	1	0	6

### Synthesis of Results

**Figure 2:**
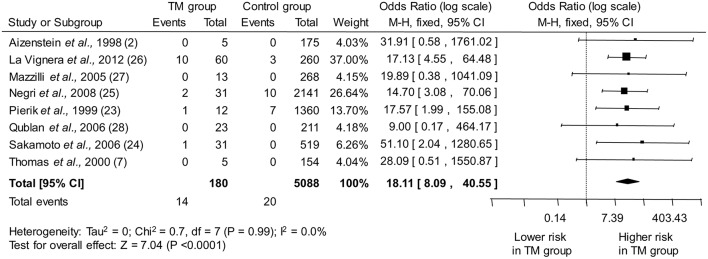
Forest plots depicting the odds ratio for testicular cancer between infertile men with and without testicular microlithiasis (TM). Diamond indicates the overall summary estimate (width of the diamond represents the 95% CI); boxes indicate the weight of individual studies in the pooled analysis. CI, confidence interval; df, degrees of freedom; M-H, Mantel-Haenszel.

The eight studies included in the meta-analysis collectively provided information on 180 infertile men with TM and 5,088 infertile men without TM. As shown in Figure 2, pooled estimate indicated that the presence of TM is associated with a ~18-fold higher odd for testicular cancer (OR: 18.11, 95% CI: 8.09, 40.55; *P* < 0.0001). No heterogeneity among the studies was observed (*P*_for heterogeneity_ = 0.99, *I*^2^ = 0%).

**Figure 3:**
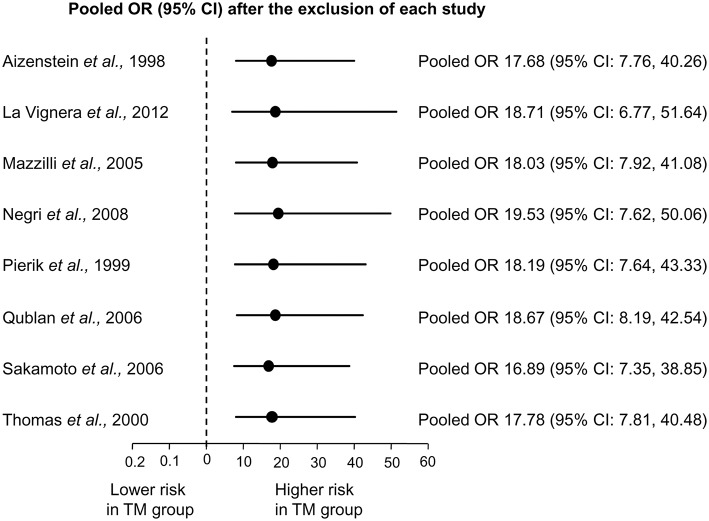
Sensitivity analysis showing the influence of each individual study on the pooled odds ratio (OR) with 95% Confidence Interval (CI) for testicular cancer.

Sensitivity analysis was performed to assess the contribution of individual studies to the overall odd for testicular cancer. As shown in Figure 3, similar pooled ORs and 95% CIs were generated with the exclusion of each study, thus indicating the high degree of stability of the results.

### Publication Bias

**Figure 4:**
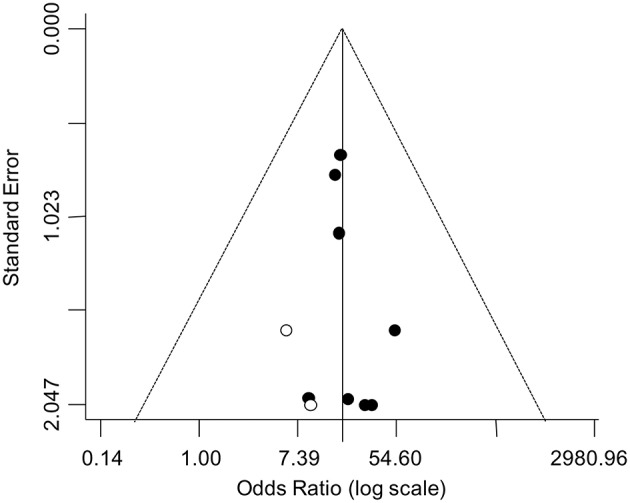
Funnel plot for the analysis of the relationship between TM and testicular cancer in infertile men. The trim-and-fill analysis identified two putative missing studies (white circle) on the left side of the distribution.

The asymmetry of the funnel plot suggested a possible publication bias (Figure 4). Accordingly, the trim-and-fill analysis identified two putative missing studies on the left side of the distribution. Nevertheless, when the funnel distribution was rebalanced by including these additional studies, the adjusted pooled estimate indicated a persistent significantly higher odd for testicular cancer in the TM group (adjusted OR: 16.42, 95% CI: 7.62, 35.37; *P* < 0.0001) with no heterogeneity (*P*_for heterogeneity_ = 0.99, *I*^2^ = 0%).

## Discussion

To date, inconclusive results have been reported by studies evaluating the association of TM with a higher prevalence of testicular cancer in infertile males. Many studies retrospectively explored the relationship between TM and testicular cancer in heterogeneous series of patients referred for scrotal US due to different urological/andrological indications, also including (but not restricted to) infertility (6, 13, 15, 35–40); in most cases, data from individual sub-group of patients were not provided separately, thus making impossible to draw conclusions regarding infertile males. Some of these studies were meta-analyzed by Wang et al. (17), reporting a strong overall association of TM with an almost 13-fold increased risk of testicular cancer. In that analysis, a very large between-studies heterogeneity was found (*I*^2^ = 82.1%) and, interestingly, the studies with the most significant forest plot results (14, 16, 26, 41) included infertile patients in their samples. Actually, even the few studies providing information about the association between TM and testicular cancer in male infertility specifically, did not produce unequivocal results.

In the largest retrospective studies, by Pierik et al. (23) and by Negri et al. (25), that enrolled only infertile men, patients with TM, who represented approximately 1% of the study populations, exhibited significantly higher odds for testicular cancer when compared to TM-free infertile patients. A significantly higher association between TM and testicular cancer was also found by Sakamoto et al. (24) and by La Vignera et al. (26) in their infertile sub-group of patients, who showed TM in 5.6 and 18.8% of cases, respectively. However, in four studies, enrolling smaller-sized infertile men samples, where the prevalence of TM ranged from 2.8 to 9.8%, no cases of testicular cancer were detected in either patients with or without TM (2, 7, 27, 28).

In the present meta-analysis of these eight carefully selected studies, the overall prevalence of testicular cancer in infertile men with TM was 7.8%, corresponding to an odd of detecting a testicular cancer approximately 18-fold higher (pooled OR : 18.11, 95% CI: 8.09, 40.55) than in infertile men without TM. Given the retrospective design of the included studies, whether TM has to be regarded as a precursor of malignant disease or whether it develops as a result of malignant disease remains uncertain.

It is generally accepted that virtually all TGCTs arise from a CIS (42), the common precursor which eventually progresses to invasive cancer if not treated. Interestingly, in a study by von Eckardstein et al. (3), a CIS was diagnosed in 2 out 11 men with TM, whereas no CIS was found in biopsies from 65 individuals without TM. The cross-sectional association between TM and CIS was subsequently confirmed by De Gouveia et al. (10) on a larger retrospective series of 263 subfertile men. Although longitudinal studies are lacking, these data suggest that, as TM has a significant predictive value for the presence of CIS (which is a precursor of TGCT), TM might also predict the development of overt testicular cancer.

It has been hypothesized that the complex relationship linking together CIS/testicular cancer, poor semen quality and closely related conditions, such as cryptorchidism and hypospadias, could reflect the testicular dysgenesis syndrome, a common underlying entity with an origin in fetal life (20). Events involved in the development of a testicular cancer in adult life are likely to occur during embryogenesis and CIS cells, which closely resemble fetal gonocytes both morphologically and immunochemically (43–45), are presumed to derive from primitive primordial germ cells or gonocytes that escaped normal differentiation *in utero*, and instead entered a neoplastic transformation (46). Similarly, any disturbance in early fetal life of the development/differentiation of Leydig and Sertoli cells may lead to an impairment of both production of testosterone and insulin-like factor 3 (INSL3) and germ cell development, resulting in genital malformations (such as hypospadias and cryptorchidism) and, later in life, impaired spermatogenesis (47). The model of the testicular dysgenesis syndrome not only could explain why infertility represents a risk factor for testicular cancer but also why the presence of TM is associated with an even higher risk: TM might be the expression of an already existent CIS.

## Limitations

Some limitations of this meta-analysis, other than the aforementioned retrospective design of the included studies, have to be recognized. Firstly, overall, meta-analyzed studies included very few patients with TM (only 180 individuals) and very few events (only 14 testicular cancers in cases and 20 in controls) resulting in quite imprecise ORs, as indicated by their wide confidence intervals. However, it should be recalled that both TM and testicular cancer represent relatively uncommon conditions. For instance, in the aforementioned study by Hanson et al. (19), who reported a more than 10-fold increase in the risk for testicular cancer in oligozoospermic men, only 30 cases of testicular cancer were found in a large sample of 20,433 subfertile men. In any case, in spite of the low number of events registered in our quantitative synthesis, at the sensitivity analysis, similar pooled ORs and 95% CIs were generated when the studies with the highest weight in contributing in the pooled estimate (23, 25, 26) were excluded, thus indicating the very high degree of stability of the results.

As another limitation of this meta-analysis, the largely incomplete information about the occurrence of other recognized risk factors, such as testicular hypotrophy and cryptorchidism, in cases and controls (Table 1), did not allow sub-group analyses for the assessment of a further increase in the risk of cancer.

Finally, the funnel plot revealed a possible publication bias, suggesting that published studies could be a not fully representative sample of the available evidence. Nevertheless, the value of the corrected pooled OR, taking into account two putative missing studies identified by the trim-and-fill analysis, demonstrated that the publication bias did not substantially affect the overall estimate.

In conclusion, the results from the present meta-analysis indicate that, in infertile men, TM is associated to an ~18-fold higher odd of detecting testicular cancer. Longitudinal studies are warranted to elucidate whether this cross-sectional association actually reflects a higher susceptibility of infertile men with TM to develop testicular cancer over time.

## Author Contributions

AB conceived the study, participated in the assessment of the eligibility of each selected study, assessed the quality of the studies, performed the statistical analysis, and wrote the manuscript. AM and EM participated in the systematic search and in the assessment of the eligibility of the studies. SD assessed the quality of the studies. DB and CC were involved in data extraction. FF helped draft the manuscript and critically revised the manuscript. SF contributed to conception, participated in the assessment of the quality of the studies, and critically revised the manuscript. All authors read and approved the final manuscript.

### Conflict of Interest Statement

The authors declare that the research was conducted in the absence of any commercial or financial relationships that could be construed as a potential conflict of interest.
