# Supplementary material for: Testicular Cancer in Infertile Men With and Without Testicular Microlithiasis: A Systematic Review and Meta-Analysis of Case-Control Studies
Source: Front Endocrinol (Lausanne). 2019 Mar 21;10:164. doi: 10.3389/fendo.2019.00164 (PMC6437042; doi:10.3389/fendo.2019.00164)
Supplement: Supplementary file 2 [file Table_2.DOC]

**Supplementary Table 2: MOOSE Checklist for Meta-analyses of Observational Studies**

| **Item No** | **Recommendation** | **Reported section** |
| --- | --- | --- |
| Reporting of background should include | | |
| 1 | Problem definition | Introduction |
| 2 | Hypothesis statement | Introduction |
| 3 | Description of study outcome(s) | Material and methods |
| 4 | Type of exposure or intervention used | Material and methods |
| 5 | Type of study designs used | Material and methods |
| 6 | Study population | Material and methods: Inclusion and exclusion criteria |
| Reporting of search strategy should include | | |
| 7 | Qualifications of searchers (*e.g.* librarians and investigators) | Material and methods |
| 8 | Search strategy, including time period included in the synthesis and key words | Material and methods: Systematic search strategy |
| 9 | Effort to include all available studies, including contact with authors | Material and methods: Systematic search strategy and data extraction |
| 10 | Databases and registries searched | Material and methods |
| 11 | Search software used, name and version, including special features used (*e.g.* explosion) | Statistical analysis |
| 12 | Use of hand searching (*e.g.* reference lists of obtained articles) | Figure 1 |
| 13 | List of citations located and those excluded, including justification | Study selection and Figure 1 |
| 14 | Method of addressing articles published in languages other than English | NA |
| 15 | Method of handling abstracts and unpublished studies | NA |
| 16 | Description of any contact with authors | Data extraction |
| Reporting of methods should include | | |
| 17 | Description of relevance or appropriateness of studies assembled for assessing the hypothesis to be tested | Material and methods: Quality assessment |
| 18 | Rationale for the selection and coding of data (*e.g.* sound clinical principles or convenience) | Material and methods: Inclusion and exclusion criteria |
| 19 | Documentation of how data were classified and coded (*e.g.* multiple raters, blinding and interrater reliability) | Material and methods: Quality assessment |
| 20 | Assessment of confounding (*e.g.* comparability of cases and controls in studies where appropriate) | Material and methods: Quality assessment |
| 21 | Assessment of study quality, including blinding of quality assessors, stratification or regression on possible predictors of study results | Material and methods: Quality assessment |
| 22 | Assessment of heterogeneity | Material and methods: Statistical analysis |
| 23 | Description of statistical methods (*e.g.* complete description of fixed or random effects models, justification of whether the chosen models account for predictors of study results, dose-response models, or cumulative meta-analysis) in sufficient detail to be replicated | Material and methods: Statistical analysis |
| 24 | Provision of appropriate tables and graphics | Table 1, Table 2, Figures 1, 2, 3, 4 |
| Reporting of results should include | | |
| 25 | Graphic summarizing individual study estimates and overall estimate | Synthesis of results: Figure 2 |
| 26 | Table giving descriptive information for each study included | Table 1 |
| 27 | Results of sensitivity testing (*e.g.* subgroup analysis) | Figure 3 |
| 28 | Indication of statistical uncertainty of findings | See “quality of the included studies”, “synthesis of results” and “publication bias” |
| Reporting of discussion should include | | |
| 29 | Quantitative assessment of bias (*e.g.* publication bias) | Publication bias: Figure 4 |
| 30 | Justification for exclusion (*e.g.* exclusion of non-English language citations) | Figure 1 |
| 31 | Assessment of quality of included studies | Quality of included studies: Table 2 |
| Reporting of conclusions should include | | |
| 32 | Consideration of alternative explanations for observed results | Discussion |
| 33 | Generalization of the conclusions (ie, appropriate for the data presented and within the domain of the literature review) | Discussion |
| 34 | Guidelines for future research | Discussion |
| 35 | Disclosure of funding source | NA |

From: Stroup et al. (30)
